# Effects of Neighborhood Discrimination Towards Mainland Immigrants on Mental Health in Hong Kong

**DOI:** 10.3390/ijerph16061025

**Published:** 2019-03-20

**Authors:** Juan Chen, Zhonglu Li, Duoduo Xu, Xiaogang Wu

**Affiliations:** 1 Department of Applied Social Sciences, The Hong Kong Polytechnic University, GH305, 11 Yuk Choi Road, Hung Hom, Kowloon, Hong Kong SAR, China; 2 College of Psychology and Sociology, Shenzhen University, Room 517, Technology Building, 3688 Nanhai Ave., Shenzhen 518060, China; 3 Division of Social Science, Institute for Advanced Study, The Hong Kong University of Science and Technology, Room 3302, Academic Building, Clear Water Bay, Kowloon, Hong Kong SAR, China; dxu@connect.ust.hk; 4 Division of Social Science, Center for Applied Social and Economic Research (CASER), The Hong Kong University of Science and Technology, Room 3374, Academic Building, Clear Water Bay, Kowloon, Hong Kong SAR, China; sowu@ust.hk

**Keywords:** immigrants, mental health, discrimination, neighborhood, family function, HKPSSD

## Abstract

Using data from a representative sample of Chinese adults who were surveyed in the Hong Kong Panel Study of Social Dynamics (HKPSSD), we estimate the effects of neighborhood discrimination towards immigrants from Mainland China on the mental health of Chinese residents in Hong Kong. Contrary to our expectations, discrimination towards immigrants from Mainland China measured at the neighborhood level is not associated with the poor mental health of post-1997 immigrants; instead, a higher level of immigrant discrimination is associated with a lower level of psychological distress for both post-1997 Mainland immigrants and other Chinese residents in Hong Kong. A functional family also appears to be a consistent predictor of better mental health for both groups. Our findings, therefore, suggest that immigrant discrimination can signify a prejudice that leads to social distance or avoidance and that the post-1997 Mainland immigrants do not have extensive contact with other local residents in Hong Kong. Although local residents’ discriminatory attitudes may not result in aggressive behaviors that have a negative impact on newcomers’ mental health, the social distance between the immigrants and the local residents is still an issue that requires further research and practical attention.

## 1. Introduction

Although it does not reflect the fundamental values of modern society, discrimination remains widespread. “Discrimination” refers to judgements and actions initiated and maintained by individuals and institutions that systematically harm members of marginalized groups and reinforce unequal systems of power and privilege [1,2]. When people belonging to certain societal group discriminate against and exclude people outside of their group, they restrict the lives of the “outsiders” [3]. Their judgements and actions are ranged along a continuum that encompasses subtle forms of disrespect and extreme violence. The effects of discrimination are both interpersonal and structural [2,4].

The consequences of discrimination on people’s health and well-being can be detrimental. Studies have documented the negative effects of discrimination (particularly self-reported interpersonal racial/ethnic discrimination) on both physical and mental health [5]. Although discrimination can be societal and structural, research has primarily focused on the impacts of discrimination at the individual level. Exposure to discrimination and the perception of discrimination in neighborhoods are believed to contribute to a greater prevalence of mental disorders such as depression and anxiety, particularly among minorities and immigrants [6]. Still few studies have tested this theoretical hypothesis using appropriate empirical measures and research design or differentiated discrimination based on race/ethnicity versus immigrant status within a suitable context.

In this article, we attempt to fill this gap by investigating the effects of discrimination towards immigrants in Hong Kong, measured at the neighborhood level, on their mental health. We particularly focus on the post-1997 Mainland immigrants, that is, the first generation of immigrants arriving from Mainland China to Hong Kong after 1997. The study context of Hong Kong allows us to delimit the concept of immigrant discrimination from discrimination based on race or ethnicity [7,8]. The data we have gathered through the Hong Kong Panel Study of Social Dynamics (HKPSSD) enable us to address the three major obstacles to determining causation of the effects of neighborhood discrimination towards Mainland immigrants on mental health: the omitted context variable bias, the problem of simultaneity, and self-selection.

### 1.1. Discrimination and Mental Health

Although the associations between discrimination and health at all levels have been argued theoretically [9], the large and growing body of empirical research has focused on interpersonal discrimination based on self-reported experience. Self-reported experiences of discrimination are regarded as a form of psychosocial stress and an important risk factor for both mental and physical health [5]. Recent reviews and meta-analyses show that the body of research on discrimination and health has grown overwhelmingly in the last twenty years [5,10,11,12,13,14]. Consistent and strong associations have been documented between self-reported experiences of discrimination and a variety of indicators of mental health and psychological well-being, as well as physical health [13,14,15]. Cross-sectional and longitudinal studies have also found consistent associations between reports of discrimination and health outcomes across cohorts, which persist even after controlling confounding variables such as personality characteristics and other threats to validity [5]. Although the research focuses on racial/ethnic discrimination [3], there is evidence that reports of racial and non-racial discrimination have similar associations with health outcomes [14,16], suggesting that common processes may underlie both [5].

At present, few question whether or not researchers should consider discrimination in studies of health disparities [5]. Yet, despite the many studies on the relationship between discrimination and health, there remain unresolved issues in the field, which centre around three points. First, factors related to perceiving and/or reporting discrimination are the main measurements employed. One of the major issues raised in existing studies is that self-reported data are not sufficient to measure exposure to racial or other forms of discrimination [17]. Second, in most studies, discrimination and health are considered at the individual level. While certain experiences of discrimination are interpersonal and blatant, most are likely to be institutional and invisible [3]. They occur at the level of the workplace, the neighborhood, and the society as a whole; there is a wide variation in the expression and experience of racial discrimination [18,19]. Studies focusing solely on experiences that people self-report and neglecting analysis of discriminatory exposures that should be measured at the population level do not reveal the full toll of discrimination [3]. Third, existing studies remain USA-based and generally focus on the experiences of American racial/ethnic minorities [20]. In concept, method, and substance, these studies may not be applicable to the analysis of discrimination in other countries. A growing number of recent studies have taken a more global approach, drawing on data on immigrants of color and indigenous peoples from diverse countries. Emerging research also focuses on various bases of discrimination, including race/ethnicity, indigenous status, immigrant status, gender, sexuality, disability, and age [3]. Although the number of investigations has dramatically expanded, the scope of research remains narrow: studies still focus on interpersonal discrimination based on self-reported data, and scant research investigates the impacts of discrimination on bases other than race or ethnicity or beyond the individual level [3].

In recent years, immigration has become a prominent social and political issue in Europe, the U.S., and several other countries/regions and anti-immigrant sentiment keeps growing [21]. An increasing body of literature has examined attitudes towards immigrants; yet the concept of immigrant discrimination has often been used interchangeably with ethnic discrimination or prejudice against ethnic minorities, or as a label based on multi-item construct [22]. The situation reflects the reality that in the Western context it is difficult to discern discrimination based on immigration status versus race/ethnicity as the immigrant groups are predominantly racial or ethnic minorities [21]. Research attempting to delimit immigrant discrimination requires appropriate study context: the immigrants should share the same racial or ethnic characteristics of the natives so that discrimination based on race or ethnicity will not be a confounding problem [7].

### 1.2. Post-1997 Immigrants from Mainland China to Hong Kong

According to the most recent 2016 Hong Kong Population By-Census, only 60.7% of Hong Kong residents were born in Hong Kong. Almost one-third (31.0%) came from Mainland China, Macao, or Taiwan, and another 8.4% were born elsewhere [23]. A total of 760,000 new arrivals were admitted from the Mainland under the one-way permit (OWP) scheme during the period from 1997 to 2012, which constitutes a major source of Hong Kong’s population growth [24,25]. Although the number of new arrivals from Mainland China (defined as people from the Mainland who have resided in Hong Kong for less than 7 years) decreased from 266,577 in 2001 to 165,956 in 2016 and their proportion in the whole population decreased from 4.1% to 2.4%, a significant number, mainly women and children, still come to Hong Kong to reunite with their families [23,26]. In addition, Mainland–Hong Kong marriages accounted for 34.7% of the total marriages registered in Hong Kong in 2016 [27]. In 2016, there were 117,523 (4.7%) domestic households with at least one member who was a new arrival from Mainland China [23].

In this study, we define post-1997 immigrants from Mainland China (also known as, post-1997 Mainland immigrants) as the first generation of Mainland-born immigrants who arrived from the Mainland to Hong Kong after the reunification of Hong Kong with China in 1997. As immigrants from Mainland China share the ethnic characteristics of Chinese residents in Hong Kong, discrimination based on race or ethnicity is not a typical problem for these immigrants [28]. It is assumed that they will integrate into Hong Kong society easily because their ethnicity, language, and cultural heritage are the same as that of the local Hong Kong population [8,29]. Yet, despite these shared ethnic and language characteristics, post-1997 Mainland immigrants still encounter negative attitudes on the part of some Hong Kong residents. Even though many of these Hong Kong residents are first or second generation immigrants themselves, they are not welcoming to later generations following a similar path due to a number of factors. Many earlier immigrants were economic or political refugees from Mainland China [30]. Some worried that they would not be able to maintain the political and socio-cultural independence from the Mainland under the principle of “one country, two systems” after 1997. The growing number of Mainland Chinese in the Hong Kong population threatens the survival of these valued differences. An antagonistic atmosphere has become more perceptible due to the economic downturns in recent years. A growing population adds greater pressure on resources that are already very scarce in this densely populated territory, especially in the areas of housing, schools, and health care [28]. Studies and surveys indicate that post-1997 immigrants from Mainland China encounter formidable obstacles to social and economic integration into Hong Kong society, resulting in high levels of psychological stress [24,28,31,32,33]. Perceived discrimination is found to be a common experience for post-1997 Mainland immigrants and associated with depressive symptoms [32].

An investigation of discrimination towards post-1997 Mainland immigrants and mental health must take into account family factors as the purpose of these immigrants from Mainland China coming to Hong Kong under the one-way permit is to reunite with their families. Many studies conducted in the West have documented the significant association between family function and mental health. There is a strong correlation between impaired family function and depression, particularly in the case of immigrant families [34,35]. Studies of post-1997 Mainland immigrants in Hong Kong confirm that they have difficulties adapting to the culture and lifestyle of Hong Kong. They suffer from strained family relationships and a lack of social support, which affect their mental health [35,36]. Still there is evidence to support the family strengths perspective, which maintains that there is a positive relationship between family dynamics and migrant adaptation [37,38,39]. These findings highlight the central importance of family as an emotional resource in times of adversity and as a protective screen against mental distress [28,40]. In the present study, we therefore control for family function when estimating the potentially detrimental consequences of immigrant discrimination measured at the neighborhood level on individual mental health status. We also look into the interaction of immigrant discrimination and the functionality of their family in order to determine the potentially protective effects of a well-functioning family.

### 1.3. Measuring Neighborhood Effects

Broadly referred to as “neighborhood effects,” neighborhood-level factors often have profound effects on the health of individuals [41,42,43]. It is generally observed that cities are characterized by dramatic inequalities in neighborhood socio-economic status (SES), built environment, social organization, and collective efficacy, which, along with other physical and social attributes, lead to variability in health outcomes among residents [6,44,45]. Studies of the mental health of immigrants further indicate that neighborhood racial/ethnic density plays an important role in early-life social interactions, psychological development, and subsequent mental health outcomes [6,46]. For instance, studies show that immigrants from non-Western countries who moved to Western countries at an early age present higher rates of schizophrenia than their non-immigrant counterparts. However, immigrants living in ethnically dense neighborhoods during adolescence appear to be protected against the risk of serious mental illness [46,47]. These findings suggest that discriminatory exposures and perceived discrimination throughout life, particularly among minorities and immigrants, can be pathways to serious mental illnesses, as well as mental disorders such as depression and anxiety [6].

While studies identify neighborhood racial/ethnic composition and socio-demographic characteristics as key factors in predicting experiences of racial discrimination, interpersonal interactions, and consequent health outcomes [18], their reliance on individual-level data makes it difficult to discover causal neighborhood effects [48]. There are three major issues that have thwarted the identification of causal relations when estimating neighborhood effects: the omitted context variable bias, the simultaneity problem, and self-selection [49]. The omitted context variable bias occurs when a key explanatory variable is not available in the dataset used for the analysis. In such cases, other variables in the model serve as statistical proxies for the missing variable and pick up the effect. While one way to address the omitted variable bias is to consider which data and variables to collect on the basis of explicit theories and hypotheses, we have to acknowledge that there will always be relevant factors not covered during the data collection process. The simultaneity problem arises from the fact that measures of neighborhood characteristics are not independent from those individuals living in the neighbourhood. An empirical solution is to use longitudinal data that will associate neighbourhood characteristics from a previous point in time to present outcomes. The issue of self-selection arises from the fact that households do not select their neighborhood at random, which leads to the endogenous membership problem. To counteract the problem of self-selection bias, researchers sometimes rely on experimental or quasi-experimental data instead of interviews or observational data [48].

Using data from a representative sample of Chinese adults in the Hong Kong Panel Study of Social Dynamics (HKPSSD), we attempt to address the three problems identified above. To reduce the omitted context variable bias, we include the percentage of new arrivals and the neighborhood socio-economic status in the model estimation as control variables of neighborhood-level contextual factors. To avoid the simultaneity problem, we use longitudinal data and census data to construct the neighborhood-level measures. To overcome self-selection, we categorized the analysis according to housing type: the public housing allocation in Hong Kong is primarily based on queuing and lottery mechanism rather than applicants’ preference or price competition, which provides a natural quasi-experimental design to rigorously test the neighbourhood effects on individual outcomes [50].

## 2. Data, Measures, and Analytical Strategy

### 2.1. The Hong Kong Panel Study of Social Dynamics

We analyse data from the Hong Kong Panel Study of Social Dynamics (HKPSSD), a city-wide representative household panel survey tracking socio-economic changes and their impact on people’s livelihood in Hong Kong. Hong Kong is divided into 18 districts, each with a district council (DC), and 405 constituency areas (DCCAs). The HKPSSD used the geo-coding scheme of 18 districts and 405 DCCAs for the sampling design, as the number of spatial units is suitable and statistical information on the demographic and housing structure at DCCA levels is readily available from the Hong Kong government [51].

Both the questionnaire design and the survey implementation of HKPSSD used the computer-assisted personal interviewing (CAPI) system and its web support system. When conducting the fieldwork, the same strict procedures for data quality control and progress monitoring were undertaken for all three waves. All interviewers received a two-day training to familiarize with the questionnaire contents and the CAPI system. They were assessed by the project research staff before they started the fieldwork. CAPI and its web support system allows fieldwork supervisors and research staff to monitor the progress of fieldwork, check for sample bias, and assess data quality simultaneously. Any abnormal patterns or problems were immediately followed up. After the fieldwork interviews, data checking, editing, and recoding were carried out to ensure a clean, consistent, and accurate database for use [51,52].

The first wave of the HKPSSD survey was successfully completed in 2011, with 3214 households, 7218 adults, and 958 children interviewed. The second wave was completed in 2013, with 2165 households, 4270 adults, and 623 children re-interviewed. A refreshment sample of 1007 households (including 1960 adults and 145 children) was added in 2014, which, together with the sample for the second wave, formed the sample for a third wave of the survey in 2015, resulting in 2404 households, 5160 adults, and 477 children. The tracking rate was 71.6% at the household level and 85.1% at the individual level [51]. All subjects gave their informed consent for inclusion before they participated in the study. The study was conducted in accordance with the Declaration of Helsinki. Approvals for the ethical review of research projects involving human subjects were granted to the authors by their home universities (HSEARS20140403002 and HKUST-6001-SPPR-08).

Data from the second wave of the HKPSSD were used to construct the discrimination measures at the neighborhood level. Data on adult participants from the third wave of the HKPSSD (*n* = 5160) were used as individual level attributes for the analysis. Cross-sectional weights were generated to adjust the individuals in the study sample to the 2011 Hong Kong Population Census on key variables including sex-age group, main economic activity status, and highest level of education completed. After proper weighting, the sample is representative of the Hong Kong adult Chinese population.

### 2.2. Measures

#### 2.2.1. Immigrant Discrimination Measured at the Neighborhood Level

Using both the second wave of the HKPSSD panel sample and the refreshment sample, we measured the adult respondents’ attitudes towards immigrants from Mainland China with the Bogardus Social Distance Scale, which is a common method to measure discriminatory attitudes and prejudice [53]. The scale uses five statements to measure respondents’ attitudes toward Mainland immigrants as (1) co-workers, (2) residents in the same neighborhood, (3) next-door neighbors, (4) visitors to their house, and (5) dates of their children or relatives. A respondent who accepts statement (5) would be regarded as less prejudiced than a respondent who accepts statement (1) or any other statement on the scale. When constructing the immigrant discrimination measure, we excluded answers from respondents who belonged to the group of post-1997 immigrants from Mainland China and compiled data for 387 DCCAs with 5882 individuals from the second wave panel sample and the refreshment sample. We further excluded 96 DCCAs with fewer than 10 respondents, aggregated the averaged data at the DCCAs level, and created the immigrant discrimination measure for 291 DCCAs with answers from 5332 respondents of the second wave HKPSSD. The final scale ranges from 1–6, with higher scores indicating higher levels of discrimination or prejudice towards immigrants from Mainland China at the neighborhood level.

#### 2.2.2. Other Neighborhood Characteristics

Neighborhood SES is an index we constructed to capture the average socioeconomic status (SES) of the population in each DCCAs in Hong Kong. Based on the 2006 population by-census data, we chose four indicators to capture socioeconomic differentials of the population across neighborhoods: Proportion of households in public rental housing, proportion of households with a total monthly income of HK $30,000 or above, proportion of the population with tertiary education or above, and proportion of the population in higher status occupations (that is, managers, administrators, professionals, and associate professionals). Following the principal component analysis (PCA), the four indicators were reduced to one dimension (a single principal component), while retaining most of the information from the neighborhood-level statistics that we had assembled. The principal component was then standardized to an index so that each DCCAs was assigned a socioeconomic score ranging from 0–100 [51,52]. We were not able to obtain the exact percentage of immigrants from Mainland China after 1997 at the DCCAs level. Data from 1997–2001 indicate that immigrants coming from Mainland China through the One-Way Permit scheme accounted for 93% of Hong Kong’s total population growth [54]. We therefore use the percentage of Chinese immigrants from the Mainland, Taiwan, and Macao within the past seven years (i.e., new arrivals) at the DCCAs level retrieved from the 2011 Hong Kong Population Census as a proxy to control in the analysis.

#### 2.2.3. HSCL Psychological Distress

Mental health outcomes were assessed at Wave 3 of the HKPSSD using the Hopkins Symptom Checklist (HSCL-10), a well-known and widely used screening instrument measuring psychological distress among adolescents and adults [55,56]. The HSCL scale has been previously demonstrated to be reliable when used with Chinese Americans [57]. The Cronbach’s α was 0.85 for the study sample. We summed up the scores on the 10 items on the checklist. The total scores range from 10–40, with higher scores indicating higher psychological distress.

#### 2.2.4. General Family Functioning

General family functioning is measured as the individual level. The McMaster Family Functioning Scale, a subscale of the McMaster Family Assessment Device (FAD), was administered at Wave 3 of the HKPSSD [58,59]. Respondents used a 4-point scale to rate the respondents’ degree of agreement with 12 statements (six positive and six negative) about the real situation of their families. The validity of this scale has been tested for Chinese families in Hong Kong [60]. After the negative items were reverse scored, the Cronbach’s α of the scale was 0.78 for the study sample. A participant’s family functioning score is the sum of the 12 items, ranging from 12–48, with higher scores indicating a more functional family.

#### 2.2.5. Self-Rated Physical Health

Self-rated physical health is assessed based on the respondents’ answer to the question, “In general, how would you rate your overall physical health?” and measured on a 5-point scale, ranging from 1 (poor)–5 (excellent). The measure is used to control for potential psychosomatic and somatopsychic effects in the analysis, which are of particular concern when investigating mental health in the Chinese context [61,62,63].

#### 2.2.6. Socioeconomic Status

Measures of socioeconomic status include education (years of schooling) and occupational status (managerial, professional, and associate professional occupations, non-managerial and non-professional occupations, and currently unemployed) at the individual level, and family income (average total household monthly income (in Hong Kong dollars) in the past 12 months, transformed into natural logarithm form), household size, and housing type (dichotomously coded into public housing versus private/subsidized housing) at the household level.

#### 2.2.7. Demographic Characteristics

Demographic information includes age (years), gender (dichotomously coded with 1 indicating female), and marital status (married, never married and separated/divorced/widowed). Whether the respondent is a post-1997 immigrant from Mainland China is coded as a dichotomous variable, with 1 indicating the first generation of immigrants arriving from Mainland China to Hong Kong after 1997.

### 2.3. Analytical Strategy

Because we exclude DCCAs with fewer than 10 respondents when constructing the immigrant discrimination measure at the neighborhood level using the second wave of the HKPSSD, we lost 701 individuals residing in these DCCAs in the third wave when merging the immigrant discrimination measure with the individual-level data, leaving a sample of 4459 residing in 291 DCCAs. In addition, there were 491 cases with missing data on the individual-level variables used in the analysis (88 cases with missing on the HSCL psychological distress measure and 403 cases with missing on other variables) from the third wave of the HKPSSD adult data, leaving a sample of 3968 individuals with no missing data.

We present descriptive statistics, first for the whole sample and then by housing type, of neighborhood characteristics, individual health/mental health outcomes and socio-demographic characteristics. Weighted means or percentages and standard deviations are reported. Ordinary least squares (OLS) regressions were applied to estimate the associations of mental health with immigrant discrimination and general family function. The analysis was conducted separately for public housing residents and private/subsidized housing residents with HSCL psychological distress as the dependent variable. Four models were estimated, respectively. Model 1 is the baseline model which only includes individual characteristics. To improve the model specification, an interaction between McMaster family functioning and post-1997 immigrants from Mainland China was added in Model 2. In Model 3, we included the three neighborhood measures while controlling individual characteristics. Model 4 is the full model with the interaction of immigrant discrimination and the functionality of their family in order to determine the potentially protective effects of a well-functioning family. The weighted maximum-likelihood method was used to estimate the parameters according to which the coefficients, robust standard errors, probability levels, and Wald F statistics of the OLS regressions were calculated.

To determine whether the missing data on independent variables at the individual level (*n* = 403) were randomly distributed among the respondents without missing HSCL psychological distress (*n* = 4371), we performed the Little’s Missing Completely At Random (MCAR) Test [64]). Because the significance value was less than 0.05, we could not conclude that the data were missing completely at random. We therefore applied multiple imputation when estimating the OLS regressions using STATA 15.0 (StataCorp LLC, Colleage Station, TX, USA). We filled missing values of the independent variables at the individual level using multivariate normal regressions. The number of imputations was five. To accommodate the interaction effects in the imputation, we generated and registered interaction terms as passive variables that are functions of imputed variables. With the missing data imputed, the sample size for the OLS regressions reported was 4371.

Because our data involves two-level hierarchical structure, we also considered the Hierarchical Linear Modeling (HLM) when estimating the association of mental health measured at the individual level with immigrant discrimination measured at the neighborhood level. However, HLM typically requires at least 30 cases within each group, whereas in our data we only have around 15 cases on average within each DCCA. Nonetheless, the results from the HLM showed similar patterns with those from the OLS regressions. We therefore report the OLS regression results in the article and the HLM models can be available upon request.

## 3. Results

### 3.1. Descriptive Statistics

Table 1 provides the descriptive statistics for the whole sample and by housing type. The differences between public housing and private/subsidized housing residents are prominent on a number of measures. In terms of neighborhood characteristics, public housing residents live in neighborhoods associated with lower levels of immigrant discrimination but higher percentages of new arrivals and lower neighborhood SES. At the individual level, residents in public housing report significantly higher levels of HSCL psychological distress, a higher proportion of post-1997 immigrants from Mainland China, lower levels of self-rated physical health, lower socioeconomic status, and older age. They are also more likely to be separated, divorced, or widowed. Although residents in public housing report lower family income and larger household size, no significant difference was observed between public housing residents and private/subsidized housing residents on the McMaster Family Functioning Scale.

### 3.2. OLS Regressions among Public Housing Residents

In Table 2, we report results from the OLS regressions, which estimate the association between immigrant discrimination and HSCL psychological distress among public housing residents. Our results indicate that, for public housing residents, post-1997 immigrants from Mainland China reported significantly worse mental health status after controlling for individual and neighborhood characteristics. The coefficient increases from 0.681 in Model 1–6.380 in Model 4 after we include three interactions among immigrant discrimination, McMaster family functioning, and post-1997 Mainland immigrants.

Contrary to our expectations, immigrant discrimination was not shown to be associated with worse mental health status for post-1997 Mainland immigrants. In fact, the negative coefficient on immigrant discrimination in Model 4 indicates that in general a higher level of immigrant discrimination is associated with a lower level of HSCL psychological distress. The negative coefficient on the interaction term between immigrant discrimination and post-1997 immigrants from Mainland China further indicates that, as the level of immigrant discrimination increased, the post-1997 Mainland immigrants reported even better mental health status than other Chinese residents in Hong Kong.

According to Table 2, a functional family appears to be a consistent enhancing factor for better mental health status (i.e., lower HSCL psychological distress) throughout the models and for both post-1997 Mainland immigrants and other Hong Kong Chinese residents. Yet, the positive coefficient on the interaction term between immigrant discrimination and McMaster family functioning in Model 4 suggests that the marginal benefit of family function on mental health declines among individuals residing in neighborhoods with higher levels of immigrant discrimination.

### 3.3. OLS Regressions among Private/Subsidized Housing Residents

The OLS regressions for private/subsidized housing residents are reported in Table 3, though these results should be viewed with caution due to their self-selection bias. Although the coefficient is positive, immigrant discrimination does not appear to be a significant predictor for HSCL psychological distress for respondents in general, nor for post-1997 immigrants from Mainland China in particular. Yet, a higher percentage of new arrivals in the neighborhood is associated with a significantly lower level of psychological distress for private/subsidized housing residents. As is the case with public housing residents, a functional family contributes to better mental health for private/subsidized housing residents, particularly for post-1997 Mainland immigrants.

## 4. Discussion

The key findings of our study indicate that for public housing residents in Hong Kong, the group of post-1997 immigrants from Mainland China reported worse mental health status than other Hong Kong Chinese residents. Yet, contrary to our expectations, neighborhood discrimination towards Mainland immigrants is not associated with worse mental health for these immigrants; instead, a higher level of immigrant discrimination is associated with a lower level of psychological distress for both post-1997 Mainland immigrants and other Chinese residents in Hong Kong. The findings may suggest that neighborhood discrimination on the part of the post-1997 Mainland immigrants leads to social distance or avoidance and the post-1997 Mainland immigrants do not have intensive contact with other local residents. Unfortunately, we did not ask questions in the HKPSSD survey that can provide empirical evidence to substantiate the argument. Many studies based on self-reported experiences however have found that Mainland Chinese immigrants are often discriminated against by Hong Kong local residents (including both natives and earlier generations of immigrants) [28,32], and they may therefore perceive local people to be unfriendly and avoid interacting too much with residents in their neighborhood [29]. Residing in ethnic enclaves and avoiding interaction with natives are important survival strategies of newly arrived immigrants: immigrants living in more integrated areas tend to have more intense and wide-ranging psychological symptoms [9].

Although the local residents’ discriminatory attitudes towards immigrants may not translate directly into aggressive behaviors that negatively impact the post-1997 immigrants’ mental health status, the social distance between the new arrivals and the local residents may still cause problems that require further research and practical attention [24]. In the short term, this distance may help prevent the negative effects of discriminatory treatment on mental health outcomes, but, in the long run, it will not promote feelings of empowerment on the part of post-1997 immigrants or the integration of the immigrant population into the host society.

As studies continue to explore the negative effects of discrimination on health, they must also focus on the factors that build resilience in order to inform future interventions (Lewis et al., 2015). In the present study, we focus particularly on neighborhood discrimination towards immigrants from Mainland China in Hong Kong, while controlling for family function in the analysis. The findings of this study show that a functional family consistently enhances the mental health status of both public housing and private/subsidized housing residents, particularly for post-1997 Mainland immigrants. This result may reflect various aspects of the immigrant experience. On the one hand, family is a strong and essential support for many of the first generation of immigrants to Hong Kong after 1997, one that reduces the difficulties and stresses experienced in a new environment. Yet, studies have also shown that those who migrate for family reunification have difficulties rebuilding their social network outside their own communities. They are reluctant to expand and mobilize their social network beyond their immediate family circle. Post-1997 Mainland immigrants in particular face major obstacles to establishing friendships with other Hong Kong residents and therefore have limited friendship networks [29]. Having a highly functional family is a protective factor for mental health status, but it may also prevent immigrants from socializing with the local population and integrating into the host society.

Still, for post-1997 Mainland immigrants without strong family support, the situation is likely to be even harsher, as rebuilding their social network is not easy either within or outside their own communities. There is a pressing need for studies that examine coping strategies at the individual and family level, as well as contextual or environmental buffering factors at the neighborhood level, that reduce immigrant stress and foster resilience [5].

The context of our study is Hong Kong’s public housing. We took measures to address the omitted context variable issue, the simultaneity problem, and the self-selection bias in identifying the causal neighborhood effects. Consequently, the results provide more robust evidence regarding the effects of neighborhood discrimination towards immigrants on residents’ mental health outcomes. Nonetheless, we are still aware that the findings cannot be easily translated to other social contexts: for example, the randomized public housing allocation could not be easily replicated in places dominated by a private housing market. It is also important to note that residents living in public housing show significant differences from those residing in private/subsidized housing. To better understand and improve residents’ mental health and general well-being, and to reduce social disparities, future research should attempt to elucidate the reciprocal links between individuals and the macro environment and to explore other forms of discrimination at various levels with appropriate research design, empirical measures, and study context.

## 5. Conclusions

To conclude based on data collected from a representative sample of Chinese adults in the HKPSSD, we estimated the effects of immigrant discrimination measured at the neighborhood level on mental health among Chinese residents in Hong Kong. We particularly focused on discrimination towards immigrants from Mainland China who arrived in Hong Kong after 1997. The study context of Hong Kong allowed us to eliminate the confounding effect of discrimination based on race/ethnicity. The data from HKPSSD enabled us to tackle the three major obstacles to the identification of causal relations when estimating the neighborhood effects: the omitted context variable bias, the simultaneity problem, and self-selection. Although research documenting the adverse effects of discrimination on health and mental health continues to grow in recent years, studies focusing on immigrant discrimination with appropriate empirical measures and study contexts have been lacking [5]. Our research effort is, therefore, critically important for the purpose of understanding and improving the mental health status and general well-being in societies shaped by numerous forms of discrimination at multiple levels.

## Figures and Tables

**Table 1:** Descriptive Statistics of the Whole Sample and by Housing Type

	Whole Sample	Public Housing	Private/Subsidized Housing	*t* Test
**Neighborhood characteristics**				
Immigrant discrimination (1–6, mean)	1.783	1.683	1.846	−0.149 ***
	(0.573)	(0.487)	(0.613)	
Percentage of new arrivals (0.4–20.8, mean)	3.797	4.285	3.488	0.779 ***
	(2.878)	(2.698)	(2.945)	
Neighborhood SES (0–100, mean)	31.901	14.179	43.134	−29.189 ***
	(20.953)	(12.436)	(17.148)	
**Individual characteristics**				
HSCL psychological distress (10–40, mean)	11.410	11.765	11.185	0.722 ***
	(2.845)	(3.420)	(2.384)	
Post-1997 immigrants from Mainland China (%)	9.697	14.177	6.857	6.069 ***
McMaster family functioning (12–48, mean)	35.789	35.874	35.735	0.011
	(4.712)	(4.812)	(4.647)	
Self-rated physical health (1–5, mean)	2.869	2.781	2.925	−0.139 ***
	(0.783)	(0.821)	(0.753)	
Years of schooling (0–22, mean)	9.951	8.688	10.752	−1.931 ***
	(4.610)	(4.589)	(4.443)	
Occupation status (%)				
Not working	40.793	47.768	36.372	9.860 ***
Non-professional/managerial occupation	43.085	45.061	41.832	2.806
Professional/managerial occupation	16.123	7.172	21.796	−12.67 ***
Family income (100–500,000, mean)	27,365.3	19,613.5	32,278.8	−12,324.9 ***
	(21,934.6)	(13,664.4)	(24,609.7)	
Ln (Family income) (4.6–13.1, mean)	9.717	9.245	10.017	−0.772 ***
	(1.493)	(1.721)	(1.237)	
Household size (1–12, mean)	3.286	3.399	3.215	0.102 *
	(1.432)	(1.398)	(1.449)	
Age (16–104, mean)	46.413	47.500	45.724	1.913 **
	(17.650)	(19.085)	(16.644)	
Gender (female, %)	54.154	55.814	53.102	1.099
Marital status (%)				
Married	59.171	53.594	62.705	−10.108 ***
Never married	30.477	32.333	29.300	3.427 *
Separated/divorced/widowed	10.353	14.074	7.994	6.681 ***
Weighted percentage	100	38.80	61.20	
Sample *n*	3968	1516	2452	

Notes: Data are weighted. Means or percentages are reported; standard deviations in parentheses. *t* tests are conducted between public housing vs. private/subsidized housing; * *p* < 0.05, ** *p* < 0.01, *** *p* < 0.001.

**Table 2:** OLS Estimating Association between Immigrant Discrimination and HSCL Psychological Distress among Public Housing Residents with Imputation.

	Model 1	Model 2	Model 3	Model 4
**Neighborhood characteristics**				
Immigrant discrimination			−0.202	−2.757 *
			(0.146)	(1.105)
Immigrant discrimination				−0.810 +
x Post-1997 immigrants from Mainland China				(0.486)
Immigrant discrimination				0.075 *
x McMaster family functioning				(0.030)
Percentage of new arrivals			2.547	3.319
			(3.736)	(3.806)
Neighborhood SES			−0.004	−0.004
			(0.006)	(0.006)
**Individual characteristics**				
Post-1997 immigrants from Mainland China	0.681 *	4.838	4.774	6.380 +
	(0.312)	(2.954)	(2.949)	(3.515)
McMaster family functioning	−0.090 ***	−0.077 ***	−0.078 ***	−0.203 ***
	(0.018)	(0.017)	(0.018)	(0.054)
McMaster family functioning		−0.116	−0.114	−0.122
x Post-1997 immigrants from Mainland China		(0.078)	(0.078)	(0.081)
Self-rated physical health	−0.823 ***	−0.827 ***	−0.825 ***	−0.811 ***
	(0.165)	(0.164)	(0.164)	(0.162)
Years of schooling	−0.005	−0.003	−0.002	−0.001
	(0.035)	(0.034)	(0.034)	(0.034)
Occupation status (ref: not working)				
Non-professional/managerial occupation	−0.412 *	−0.413 *	−0.411 *	−0.409 *
	(0.176)	(0.176)	(0.176)	(0.177)
Professional/managerial occupation	−0.321	−0.308	−0.283	−0.303
	(0.297)	(0.297)	(0.300)	(0.300)
Ln (Family income)	−0.217 *	−0.223 *	−0.225 *	−0.231 *
	(0.091)	(0.090)	(0.090)	(0.089)
Household size	−0.058	−0.057	−0.053	−0.044
	(0.075)	(0.075)	(0.076)	(0.075)
Age	0.016 +	0.016 +	0.017 *	0.017 *
	(0.009)	(0.009)	(0.008)	(0.008)
Female	0.209	0.199	0.197	0.178
	(0.168)	(0.168)	(0.169)	(0.170)
Marital status (ref: Married)				
Never married	0.436 +	0.425 +	0.439 +	0.449 +
	(0.238)	(0.239)	(0.237)	(0.235)
Separated/divorced/widowed	0.305	0.316	0.306	0.319
	(0.326)	(0.325)	(0.324)	(0.322)
Constant	18.584 ***	18.169 ***	18.453 ***	22.639 ***
	(1.452)	(1.378)	(1.416)	(2.399)
Sample *n*	1649	1649	1649	1649
Wald F Statistics	13.859	12.769	10.765	9.515

Notes: Robust standard errors in parentheses; *** *p* < 0.001, ** *p* < 0.01, * *p* < 0.05, + *p* < 0.10.

**Table 3:** OLS Estimating Association between Immigrant Discrimination and HSCL Psychological Distress among Private/Subsided Housing Residents with Imputation.

	Model 1	Model 2	Model 3	Model 4
**Neighborhood characteristics**				
Immigrant discrimination			0.153 +	0.127
			(0.080)	(0.657)
Immigrant discrimination				0.380
x Post-1997 immigrants from Mainland China				(0.378)
Immigrant discrimination				−0.000
x McMaster family functioning				(0.019)
Percentage of new arrivals			−6.069 ***	−6.137 ***
			(1.394)	(1.410)
Neighborhood SES			−0.004 +	−0.005 +
			(0.003)	(0.003)
**Individual characteristics**				
Post-1997 immigrants from Mainland China	0.056	3.047	3.215 +	2.405
	(0.191)	(1.884)	(1.839)	(1.807)
McMaster family functioning	−0.055 ***	−0.049 ***	−0.048 ***	−0.048
	(0.012)	(0.012)	(0.013)	(0.035)
McMaster family functioning		−0.084 +	−0.088 +	−0.085 +
x Post-1997 immigrants from Mainland China		(0.050)	(0.049)	(0.048)
Self-rated physical health	−0.192 *	−0.197 *	−0.198 *	−0.201 **
	(0.078)	(0.078)	(0.077)	(0.077)
Years of schooling	0.009	0.008	0.009	0.008
	(0.018)	(0.018)	(0.018)	(0.018)
Occupation status (ref: not working)				
Non-professional/managerial occupation	−0.043	−0.039	−0.050	−0.050
	(0.118)	(0.118)	(0.119)	(0.119)
Professional/managerial occupation	0.028	0.035	0.013	0.017
	(0.162)	(0.163)	(0.163)	(0.164)
Ln (Family income)	−0.166 *	−0.165 *	−0.163 *	−0.160 *
	(0.070)	(0.069)	(0.070)	(0.069)
Household size	−0.040	−0.043	−0.050	−0.050
	(0.032)	(0.032)	(0.032)	(0.032)
Age	0.001	0.001	0.001	0.001
	(0.005)	(0.005)	(0.005)	(0.005)
Female	0.126	0.126	0.124	0.127
	(0.103)	(0.103)	(0.103)	(0.103)
Marital status (ref: married)				
Never married	0.316 *	0.322 *	0.316 *	0.315 *
	(0.142)	(0.142)	(0.141)	(0.141)
Separated/divorced/widowed	1.231 ***	1.241 ***	1.209 ***	1.202 ***
	(0.270)	(0.270)	(0.268)	(0.267)
Constant	15.122 ***	14.939 ***	14.993 ***	15.062 ***
	(1.006)	(1.001)	(1.047)	(1.389)
Sample *n*	2722	2722	2722	2722
Wald F statistics	7.621	7.109	6.991	6.404

Notes: Robust standard errors in parentheses; *** *p* < 0.001, ** *p* < 0.01, * *p* < 0.05, + *p* < 0.10.
